# Focusing on individual morphological fracture characteristics of pelvic ring fractures in elderly patients can support clinical decision making

**DOI:** 10.1186/s12877-022-03222-0

**Published:** 2022-06-30

**Authors:** Michaela Ramser, Dieter Cadosch, Werner Vach, Nathalie Strub, Franziska Saxer, Henrik Eckardt

**Affiliations:** 1 University Hospital Basel, Department of Orthopaedic and Traumatology Surgery, 4031 Basel, Basel, Switzerland; 2 University of Basel, Basel, Switzerland; 3 Basel Academy for Quality and Research in Medicine, Basel, Switzerland; 4 Novartis Institutes for Biomedical Research, Basel, Switzerland

**Keywords:** Pelvic fracture, Frailty, Elderly, Outcome, Surgery, Conservative treatment, Failure of conservative treatment mortality, Individualised care, Patient-centred treatment, Fracture characteristics, Fragility fractures of the pelvis, FFP

## Abstract

**Introduction:**

Pelvic ring fractures in the elderly are often caused by minor trauma. Treatment of these patients is currently based on fracture classification, clinical course, and ability to mobilize. Our aim was to identify morphological fracture characteristics with potential prognostic relevance and evaluate their association with clinical decision making and outcome, as well as their interobserver reliability.

**Methods:**

Five fracture characteristics were investigated as potential variables: 1. Extent of the dorsal pelvic ring fracture (absent, unilateral, bilateral); 2. Extent of the ventral pelvic ring fracture (absent, unilateral, bilateral); 3. Presence of a horizontal sacral fracture; 4. Ventral dislocation; 5. Ventral comminution.

These characteristics were assessed retrospectively in a series of 548 patients. The association of their presence with the decision to perform surgery, failure of conservative treatment and the length of hospital stay (LOS) was determined. Further, the inter-observer reliability for the specific characteristics was calculated and the relation with survival assessed.

**Results:**

Four of the five evaluated characteristics showed an association with clinical decision making and patient management. In particular the extent of the dorsal fractures (absent vs. unilateral vs. bilateral) (OR = 7.0; *p* < 00.1) and the presence of ventral comminution/dislocation (OR = 2.4; *p* = 0.004) were independent factors for the decision to perform surgery. Both the extent of the dorsal fracture (OR = 1.8; *p* < 0.001) and the presence of ventral dislocation (OR = 1.7; *p* = 0.003) were independently associated with a prolonged overall LOS.

The inter-observer agreement for the fracture characteristics ranged from moderate to substantial.

A relevant association with increased mortality was shown for horizontal sacral and comminuted ventral fractures with hazard ratios (HR) of 1.7 (95% CI: 1.1, 2.5; *p* = 0.008) and HR = 1.5 (95% CI: 1.0, 2.2; *p* = 0.048).

**Conclusion:**

In the elderly, the extent of the dorsal fractures and the presence of ventral comminution/dislocation were associated to the decision to undergo surgery, failure of conservative treatment and length of stay. Survival was related to horizontal sacral fractures and ventrally comminuted fractures. These characteristics thus represent a simplified but highly informative approach for the evaluation of pelvic ring fractures in the elderly. This approach can support clinical decision making, promote patient-centred treatment algorithms and thus improve the outcome of individualized care.

**Supplementary Information:**

The online version contains supplementary material available at 10.1186/s12877-022-03222-0.

## Introduction

Pelvic ring fractures in elderly patients are a growing public health problem because of their impact on individuals and their increasing incidence with the aging of society [1–3]. Overall, reported one-year mortality after a pelvic ring fracture in the elderly ranges from 11 to 27% [4–12].

Pelvic ring fractures are associated with low-energy trauma, such as a fall from a sitting or standing position, and have recently been established as a separate diagnostic entity [13, 14].

Fracture patterns and associated injuries differ significantly from the features seen in younger patients after high-energy trauma [15–17]. To better capture the fracture patterns observed in the elderly Rommens and Hoffmann propose a separate fracture classification for these fragility fractures of the pelvis (FFP) based on computed tomography (CT) [14]. Basically, the fractures are divided into 4 main categories reflecting an increasing degree of instability. In addition, the main categories are divided into subgroups reflecting the location and extent of the dorsal fracture component.

The treatment approach in the young and elderly is different, especially because pelvic ring fractures in the elderly are mostly stable due to intact ligamentous structures [18]. Surgery to stabilize the pelvic ring is recommended by some authors for a selected group of patients with bilateral or displaced dorsal fractures [14]. However, comparative studies evaluating surgery vs conservative treatment for these fractures are largely missing and treatment approaches are highly individualized. In recent years, there has been increasing recognition in research and clinical practice that frailty has an impact on treatment outcomes in older patients. Consequently, aspects such as baseline mobility, independence, treatment expectations, comorbidities, etc. must be incorporated into treatment algorithms. Most pelvic ring fractures in the elderly can initially be treated conservatively with analgesic, physical therapy and supported mobilisation [13, 19–23]. If conservative treatment proves unsuccessful and mobilisation is associated with persistent pain, surgical stabilisation of the fracture should be considered, optimally within 2 weeks after trauma to avoid immobility-associated decline [14, 23, 24]. However, it must be acknowledged that there is a high variation in treatment algorithms across institutions and countries and a general consensus is lacking. The algorithms reflect clinical reasoning and institutional experience [13, 24–27]. We may, however, see some consensus that an individualized treatment approach is desirable. There is a plethora of patient factors that may play a role during the discussion of treatment options, but fracture morphology is a central aspect. The need for an individualized treatment approach may also explain the lack of comparative intervention studies in this patient group.

We aimed to identify fracture characteristic with a potential prognostic value and to explore the properties of these distinct fracture characteristics with respect to their potential impact on clinical decision making, patient management and outcome. We considered fracture characteristics of low-energy pelvic ring fractures which are overall also reflected in the FFP classification but so far not evaluated individually: the extent of the dorsal and ventral aspect of the pelvic ring fracture (absent, unilateral, bilateral), the presence/absence of a horizontal sacral fracture and the presence/absence of comminution and/or dislocation of the ventral fracture.

## Methods

### Regulatory framework

The study was approved by the competent ethical committee “Ethikkommission Nordwest- und Zentralschweiz; EKNZ” (Ref. 2017-01859, ClinicalTrials.gov Identifier: NCT03476824). All methods were performed in accordance with the relevant guidelines and regulation. It followed applicable law as well as good clinical practice (GCP) and the Declaration of Helsinki.

### Patient population

All patients ≥60 years old treated for a low-energy pelvic ring fracture at our hospital between January 2006 and December 2018 were included unless a dissent for the use of routine data was documented. Patients were identified by screening the electronic patients’ records for the following keywords: pelvic ring fracture, pelvic fracture, ala fracture, sacrum fracture and ramus pubis fracture. We also screened for acetabular fractures in order not to miss fractures of the anterior wall as extension of a pubic ramus fracture. The documented injury mechanism allowed a discrimination between patients after low- or high-energy injury according to a predetermined list of possible trauma mechanisms. Patients with high-energy trauma like traffic accidents, bicycle accidents, falls on stairways or falls from considerable height other than standing position were excluded.

### Data collection

Basic demographic information was extracted from the patient records at baseline. In addition, the three components of the Parker Mobility Score (PMS) [28] (able to walk inside the house; able to walk outside the house; able to go shopping, to a restaurant or to visit the family) and the Elixhauser Comorbidity Index (ECI) [29] were assessed based on documented anamnestic information and comorbidities, reflecting solicited information from patients, their families, primary care physicians or nursing institutions.

Patient records were also the source for data on the performance of surgery, the length of stay (LOS) and discharge management. If a second hospitalisation occurred for either further analgetic treatment in the course of conservative treatment or secondary surgical fixation of the fracture after failed conservative treatment, the same data set was extracted.

### Fracture characteristics

A group of experienced surgeons identified five fracture characteristics of FFP that are frequently encountered when analysing these fractures, that were regarded as relevant and well defined. Most of these fracture characteristics are key features of the established FFP classification and solely or in a combination represented there [14]. Only the more detailed assessment of the ventral fracture with an additional evaluation of dislocation and comminution was added as separate characteristics, as both features could potentially be an indirect indication of instability to simple ventral fractures. The five characteristics are:

- Extent of the dorsal fractures: absent, unilateral, bilateral
- Extent of the ventral fractures: absent, unilateral, bilateral
- Horizontal sacral fracture: absent or present
- Dislocated ventral fracture (more than half of the bone diameter in any direction): absent or present
- Comminuted ventral fracture: absent or present

The “extent” in this context refers to the spatial distribution of osseous damage (either absent, unilateral fracture or bilateral fracture) and not to the degree of disruption e.g., complete vs. incomplete, nor the exact location e.g., sacral vs. iliacal which is not considered.

The order of the above listed main categories does not reflect a hierarchical order of fracture severity or pelvic instability.

### Evaluation of CT scans

All radiological examinations performed at the first presentation were retrospectively assessed.

For the assessment of interobserver agreement of the fracture characteristics only patients treated between January 2017 and December 2018 were included. Each CT scan was classified independently by four surgeons who had access to the definition of the above-described fracture characteristics and the definitions of the established FFP classification at all time. However, in this sub-study the observers did not distinguish between comminuted or dislocated fractures.

All observers were blinded for any demographic or clinical information including treatment (operative vs. conservative).

### Decision for surgery

**Fig. 1:**
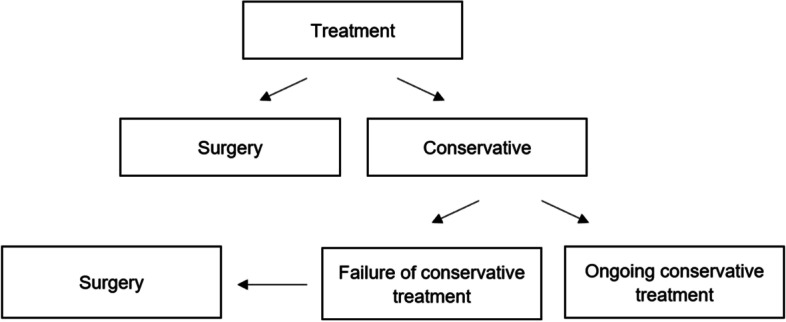
Decision making for the treatment of pelvic fractures in the elderly

The overall decision for surgery (i.e., the indication for surgery by the treating surgical team and the acceptance of the treatment proposition by the patient) were divided into individual decisional pathways along the treatment course and separately evaluated. Consequently, three variables were considered (Fig. 1):

- Surgery during the initial hospitalisation vs. conservative treatment
- Rehospitalisation for surgery after failure of conservative treatment vs. continuation of conservative treatment
- Overall decision for surgery (early or late) vs. conservative treatment

### Length of hospital stay

With respect to LOS three variants are considered: the overall length of all hospitalizations, the overall length of the first hospitalization and the length of the first hospitalisation in conservatively treated patients.

### Survival analysis

Survival analysis was performed in the conservatively treated patients to avoid a confounding effect from surgery.

Survival information for patients was available via the registration office or the national pension scheme. The end of the observation period (January 2020) was regarded as censoring events.

### Statistical methods

For the description of patient characteristics arithmetic means and the interquartile range (IQR) are presented for continuous variables, categorial data are reported as absolute and relative frequencies.

The association between fracture characteristic and the decision for surgery is visualised by bar charts and quantified by odds ratios based on logistic regression. To assess the independent value of the fracture characteristics as risk factors for surgery we fit logistic regression models with all five characteristics and PMS and ECI as covariates and report adjusted odds ratios. Missing values in the PMS were handled by the missing indicator approach. For analysing the LOS, we use the categories 0, 1, 2-6, 7-13, 14-20, and ≥ 21 days. The association between fracture characteristics and the LOS is visualized by stacked bar charts and quantified by odds ratios from an ordinal regression model. This model is also used to assess the independent value of each characteristic by reporting adjusted coefficients. LOS was not analysed as a continuous outcome due to a bimodal distribution. The extent of dorsal or ventral fractures, respectively, was handled as a continuous covariate coded as 0, 1, and 2, implying that odds ratios refer to the move from one level to the next. The association with survival was analysed using a Cox proportional hazard model.

The agreement between the observers is described by agreement rates and Cohen’s kappa. The grading system of Landis and Koch [30] to interpret the kappa values is used.

All statistical analyses were performed using STATA 16.1 (StataCorp. 2019. *Stata Statistical Software: Release 16*. College Station, TX: StataCorp LLC) and Excel (Microsoft Windows 10, Redmont, WA).

## Results

### Patient population

A total of 548 patients with a CT scan proven, low-energy pelvic ring fracture were included in this study. Patient characteristics are shown in Table 1.

**Table 1:** Patient characteristics and fracture treatment

	Overall	Conservative	Operative
	*n =* 548	*n =* 462 (84.3%)	*n =* 86 (15.7%)
Age	*(n = 548)*	*(n = 462)*	*(n = 86)*
mean (IQR)	83.2 (71.0-92.0)	83.6 (79.0-89.0)	81.0 (77.0-87.0)
60-69	40 (7.3%)	30 (6.5%)	10 (11.6%)
70-79	118 (21.5%)	96 (20.8%)	22 (25.6%)
80-89	275 (50.2%)	231 (50.0%)	44 (51.2%)
≥ 90	115 (21.0%)	105 (22.7%)	10 (11.6%)
Gender	*(n = 548)*	*(n = 462)*	*(n = 86)*
female	465 (84.9%)	389 (84.2%)	76 (88.4%)
male	83 (15.1%)	73 (15.8%)	10 (11.6%)
Living situation at baseline	*(n = 398)*	*(n = 345)*	*(n = 53)*
home alone independently	126 (31.7%)	109 (31.6%)	17 (32.1%)
home with partner independently	95 (23.9%)	75 (21.7%)	20 (37.7%)
home with support	58 (14.6%)	48 (13.9%)	10 (18.9%)
nursing home	119 (29.9%)	113 (32.8%)	6 (11.3%)
Mobility at baseline	*(n = 430)*	*(n = 356)*	*(n = 74)*
with walking aid	262 (60.9%)	218 (61.2%)	44 (59.5%)
without walking aid	168 (39.1%)	138 (38.8%)	30 (40.5%)
Parker mobility score (PMS)	*(n = 409)*	*(n = 337)*	*(n = 72)*
mean (IQR)	7.3 (6.0-9.0)	7.2 (6.0-9.0)	7.8 (6.0-9.0)
1-5	71 (17.4%)	62 (18.4%)	9 (12.5%)
6	71 (17.4%)	57 (16.9%)	15 (19.4%)
7-8	42 (10.3%)	40 (11.9%)	2 (2.8%)
9	225 (55.0%)	178 (52.8%)	47 (65.3%)
Elixhauser comorbidity index (ECI)	*(n = 548)*	*(n = 462)*	*(n = 86)*
mean (IQR)	3.0 (1.0-4.0)	3.0 (1.0-4.0)	3.0 (2.0-4.0)
0	40 (7.3%)	34 (7.4%)	6 (7.0%)
1	100 (18.2%)	85 (18.4%)	15 (17.4%)
2	110 (20.1%)	91 (19.7%)	19 (22.1%)
4-5	181 (33.0%)	155 (33.5%)	26 (30.2%)
≥ 5	117 (21.4%)	97 (21.0%)	20 (23.3%)
Place of accident	*(n = 518)*	*(n = 444)*	*(n = 74)*
indoor	381 (73.6%)	331 (74.5%)	50 (67.6%)
outdoor	137 (26.4%)	113 (25.5%)	24 (32.4%)
Length of stay 1st hospitalisation (days)	*(n = 548)*	*(n = 462)*	*(n = 86)*
same day discharge	97 (17.7%)	79 (17.1%)	18 (20.9%)
1	193 (35.2%)	185 (40.0%)	8 (9.3%)
2-6	61 (11.1%)	53 (11.5%)	8 (9.3%)
7-13	121 (22.1%)	97 (21.0%)	24 (27.9%)
14-20	51 (9.3%)	33 (7.1%)	18 (20.9%)
≥ 21	25 (4.6%)	15 (3.2%)	10 (11.6%)
Treatment	*(n = 548)*	*(n = 462)*	*(n = 86)*
conservative	462 (84.3%)	462 (100.0%)	
surgery	86 (15.7%)		86 (100.0%)
early surgery	49 (57.0%)		49 (57.0%)
late surgery after failed conservative treatment	37 (43.0%)		37 (43.0%)
Time from first presentation to early surgery (days)			*(n = 49)*
mean (IQR)			6.0 (1.0-10.0)
Time from first presentation to late surgery (days)			*(n = 37)*
mean (IQR)			38.5 (2.0-50.0)
Discharge after first presentation	*(n = 548)*	*(n = 462)*	*(n = 86)*
nursing home	65 (11.9%)	61 (13.2%)	4 (4.7%)
home independently/with support	56 (10.2%)	43 (9.3%)	13 (15.1%)
rehabilitation center	419 (76.5%)	350 (75.8%)	69 (80.2%)
inhospital mortality	4 (0.7%)	4 (0.9%)	
unknown	4 (0.7%)	4 (0.9%)	
Length of stay 2nd hospitalisation (days)	*(n = 39)*	*(n = 2)*	*(n = 37)*
2-6	5 (12.8%)		5 (13.5%)
7-13	24 (6.15%)	2 (100%)	22 (59.5%)
14-20	4 (10.3%)		4 (10.8%)
≥ 21	6 (15.4%)		6 (16.2%)

In total, 86 patients (15.7%) underwent surgery. Thereof, forty-nine patients (57%) received early surgical fixation during the initial hospitalisation, 37 patients (43%) were re-admitted for failure of the conservative treatment and persistent pain during mobilisation despite adequate analgesia (Table 1) with subsequent surgery.

Among the 462 conservatively treated patients, survival data could not be obtained from 34 patients. Hence, 428 patients were considered for the survival analysis. The median follow-up time was 59 months (range 3 days to 13.6 years), and 1 year mortality of conservatively treated patients was 18.5%. In total, 225 patients died during the course of follow-up, the in-hospital mortality was 0.7%.

The length of hospital stay (LOS) varied considerably. A large proportion of patients (52.9%) was discharged on the day of presentation or the following day. Of these, only 59 patients (10.9%) were further treated on an out-patient basis (discharge home or to nursing home), while 419 patients (76.5%) were discharged to a (geriatric) rehabilitation centre (Table 1).

The distribution of the above defined fracture characteristics and the categories/subcategories of the established FFP classification are shown in Table 2.

**Table 2:** Distribution of specific fracture characteristics in the study population

Fracture characteristics	*(n = 548)*
Dorsal fracture	
no	93 (17.0%)
unilateral	339 (61.9%)
bilateral	116 (21.2%)
Ventral fracture	
no	45 (8.2%)
unilateral	439 (80.1%)
bilateral	64 (11.7%)
Comminuted/dislocated ventral fracture	
no	347 (63.3%)
yes	201 (36.7%)
Horizontal sacral fracture	
no	443 (80.8%)
yes	105 (19.2%)

### Relation of fracture characteristics to decision for surgery

**Fig. 2:**
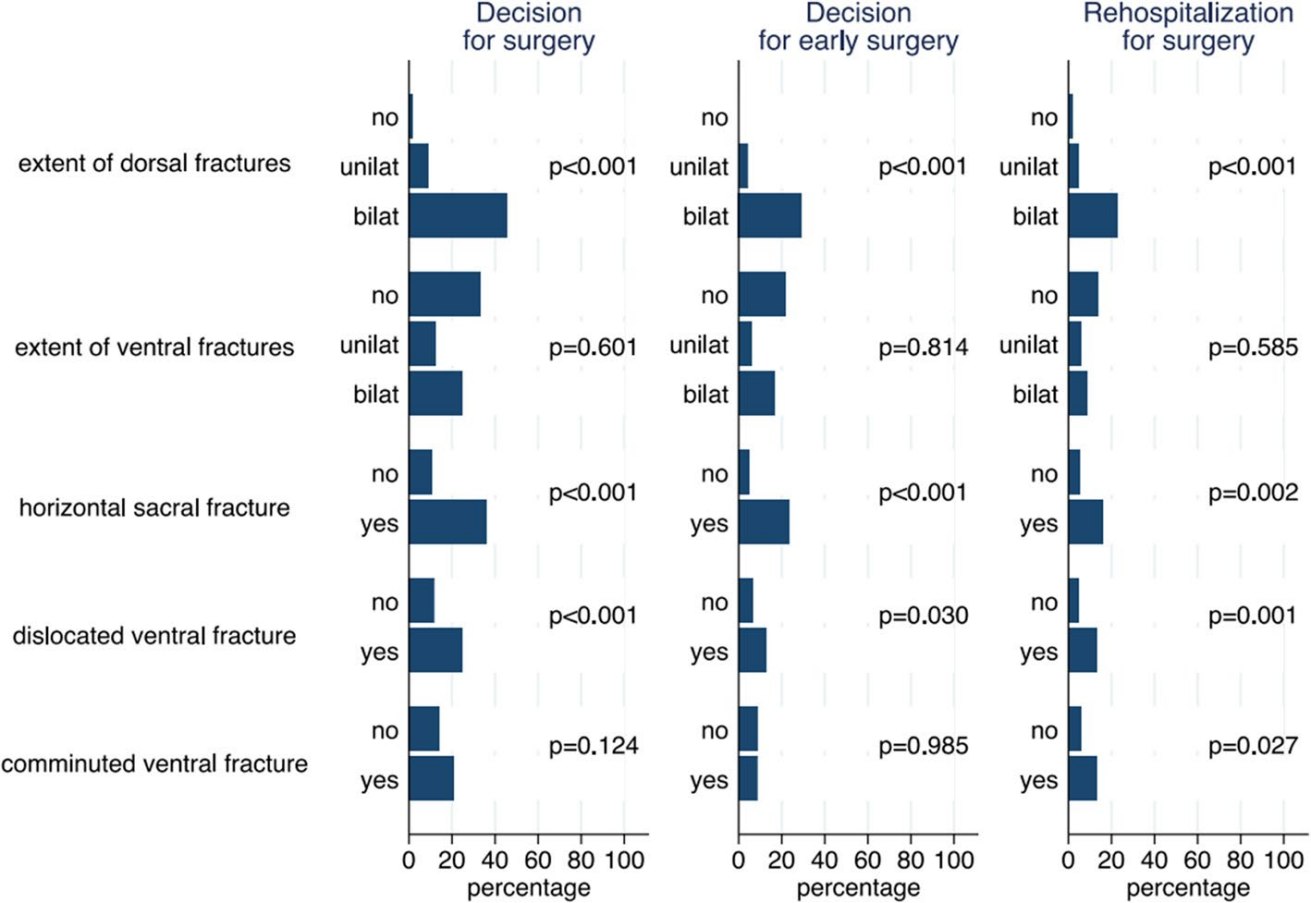
Relation of individual fracture characteristic and clinical decision-making

For three of the five fracture characteristics a distinct association with the overall decision for surgery could be observed: extent of dorsal fractures, horizontal sacral fractures, and dislocated ventral fractures (Fig. 2). Additional multivariate analyses indicate that the extent of the dorsal factures (OR = 7.0; 95% CI: 3.8-13.0; *p* < 00.1) and the presence of ventral dislocation (OR = 2.4; 95% CI: 1.3-4.2; *p* = 0.004) act as independent risk factors for surgery (Supplement 1). Therefore, with each increment in the extent of the dorsal fracture (absent, unilateral, bilateral) a 7-fold increase in the choice for surgical stabilisation was observed. For the presence of dislocation in ventral fractures the increase was 2.4-fold.

When considering the decision for early surgery or rehospitalisation for surgery, we observe similar results (Fig. 2), except that in the multivariate analyses only the extent of the dorsal fracture was identified as an independent risk factor for early surgery (OR = 8.8; 95% CI: 4.0-19.1; *p* < 0.001). Rehospitalisation for surgery due to failure of conservative treatment was associated both with the extent of dorsal fracture (OR = 4.2; 95% CI: 1.9-9.4; *p* < 0.001) and with the presence of ventral dislocation (OR = 2.70; 95% CI: 1.2-5.8; *p* = 0.015).

### Relation of fracture characteristics to the length of stay

**Fig. 3:**
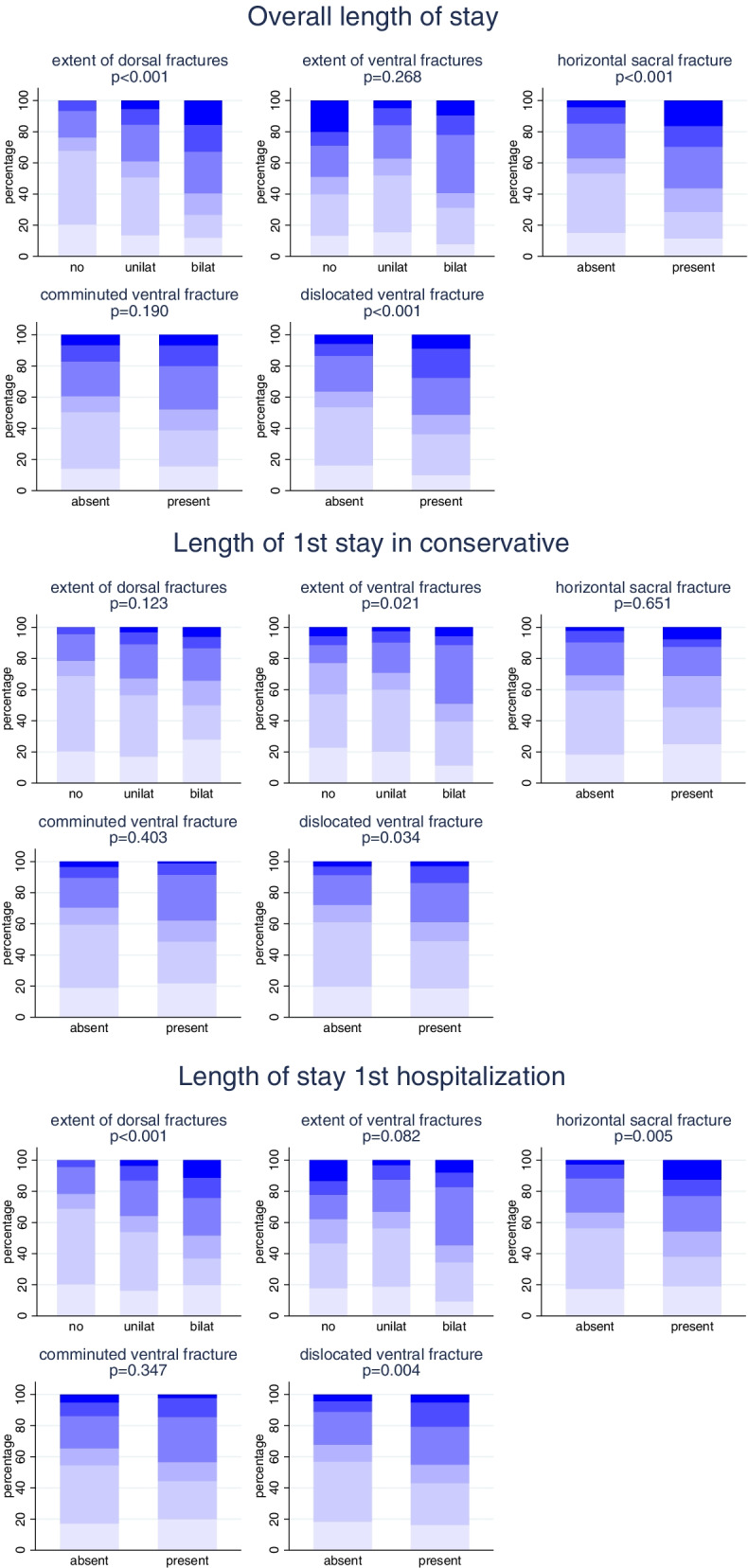
Relation of individual fracture characteristics and length of stay in surgically and conservative treated patients

Again, for three of the five fracture characteristics a distinct association with the overall LOS could be observed (Fig. 3). Both the extent of the dorsal fracture (OR = 1.8; 95% CI: 1.4-2.5; *p* < 0.001) and the presence of ventral dislocation (OR = 1.7; 95% CI: 1.2-2.4; *p* = 0.003) were independently associated with a prolonged overall LOS (Supplement 2). The association to the LOS of the first stay for conservatively treated patients was less pronounced and the strongest association was found for the extent of the ventral fracture but did not reach statistical significance.

### Relation of fracture characteristics to survival

**Fig. 4:**
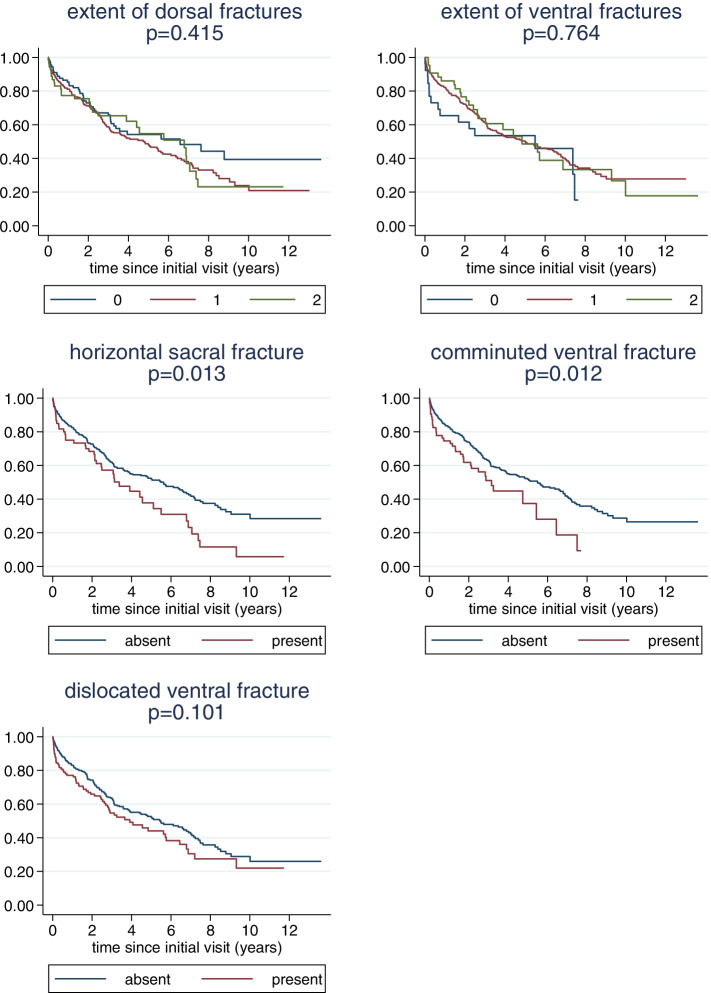
Fracture characteristic and survival

A clear relation of the extent of the dorsal or ventral fractures to overall survival could not be observed (Fig. 4). However, for the other three binary characteristics, the presence of the characteristics was always associated with worse survival, and the association was statistically significant for horizontal sacral fractures and ventral comminuted fractures. A multivariate analysis confirmed an independent prognostic value of comminuted ventral and horizontal sacral fractures with hazard ratios (HR) of 1.7 (95% CI: 1.1, 2.5; *p* = 0.008) and HR = 1.5 (95% CI: 1.0, 2.2; *p* = 0.048), respectively, indicating a relevant association with increased mortality (Supplement 3).

### Inter-observer reliability

The agreement between four surgeons with respect to the fracture characteristics is depicted in Table 3. With few exceptions, the degree of agreement expressed by the kappa values was moderate to substantial. Lower kappa values were in particular observed for the horizontal sacral fracture.

**Table 3:** Interobserver agreement in the assessment of fracture characteristics shown as kappa (κ) and agreement rates (AR)

Dorsal fracture			
	Surg1	Surg2	Surg3
Surg4	*κ* 0.60, AR 78%	*κ* 0.57, AR 77%	*κ* 0.67, AR 82%
Surg1		*κ* 0.44, AR 69%	*κ* 0.64, AR 80%
Surg2			*κ* 0.64, AR 80%
Ventral fracture			
	Surg1	Surg2	Surg3
Surg4	*κ* 0.55, AR 88%	*κ* 0.56, AR 90%	*κ* 0.43, AR 86%
Surg1		*κ* 0.48, AR 86%	*κ* 0.57, AR 88%
Surg2			*κ* 0.53, AR 89%
Horizontal sacral fracture			
	Surg1	Surg2	Surg3
Surg4	*κ* 0.60, AR 86%	*κ* 0.26, AR 84%	*κ* 0.59, AR 87%
Surg1		*κ* 0.18, AR 77%	*κ* 0.58, AR 85%
Surg2			*κ* 0.31, AR 85%
Comminution/dislocation of ventral fracture			
	Surg1	Surg2	Surg3
Surg4	*κ* 0.36, AR 69%	*κ* 0.54, AR 85%	*κ* 0.48, AR 75%
Surg1		*κ* 0.23, AR 63%	*κ* 0.54, AR 77%
Surg2			*κ* 0.32, AR 69%

## Discussion

We investigated five individual fracture characteristics of low-energy pelvic ring fracture, namely the extent of the dorsal and ventral aspect of the pelvic ring fracture, a horizontal sacral fracture and a comminution or dislocation of the ventral fracture. All characteristics showed some association with the clinical decision-making or outcome in our department.

Importantly, only patients with a defined low-energy trauma were included and all patients with a suspected higher impact excluded. A possible influence of the trauma itself on the fracture morphology, outcome or complications was thereby minimised. Two thirds of the patients were older than 80 years and still living at home. Some had additional help for tasks of daily living, needed a walking aid, but did not depend on permanent support.

In our analysis, both the extent of dorsal fracture and the presence of ventral dislocation act as independent predictors of the decision to operate and overall LOS. In contrast, the extent of ventral fracture and the presence of ventral fracture dislocation appear to be associated with prolonged LOS in conservatively treated patients, possibly due to a higher degree of pain during mobilisation due to instability at multiple sites. The extent of dorsal fracture and ventral dislocation were in addition associated with secondary surgical fixation. This group not only includes patients to whom conservative treatment was recommended, but also patients who initially refused surgery. The observed an increase in LOS depending on the extent of the ventral fracture, as well as a higher failure rate of conservative treatment in complex (comminution/dislocation) ventral fracture patterns. This finding implies that ventral pelvic ring fractures are more important for fracture-related pelvic ring instability and pain than currently believed.

Our results also suggest that certain aspects of dorsal and ventral fracture appear to have an impact on survival. It may therefore be beneficial to consider them in future decisions about treatment options for these patients. In analysing the impact on survival, we limited the analysis to patients treated conservatively to exclude the possibility that surgical intervention act as a confounding factor. We found that the presence of a horizontal sacral fracture or a ventral comminuted fracture was associated with higher mortality. This finding is in line with the increased mortality of elderly patients with an AO type B fractures compared to AO Type A fracutres after low energy trauma [31]. In contrast, the categories of the FFP classification have neither in our population (data not shown) nor in a previous study been reported to be associated with survival or mortality [32]. Neither did the alphanumeric classification by Krappinger et al. correlate to mortality [33].

Comparing the inter-observer reliability in determining the fracture characteristics between four surgeons was convincing (predominantly moderate to substantial according to Landis and Koch). Compared to similar investigations for the established FFP classification, the reliability was similar [34–36]. However, the use of fracture characteristics or FFP classifications seem to require some training in order to reduce inter-observation variation. It has to be noted, that the low-contrast representation of osteoporotic bone in CT scans makes the judgment of a fracture or the exact extent and the severity of dislocation in the pelvis challenging and difficult [36].

The three outcomes considered in this paper should be interpreted in different manners. An association between fracture characteristics and the decision for surgery reflects that these characteristics may have been taken into consideration in the decision making – and may hence play a role in the current decision making. This does not imply that it is adequate to take them into account and that they are true prognostic factors. In addition, these associations may reflect a local tradition – in other hospitals the associations may be different. In contrast, an association between fracture characteristics and mortality is a sign for a true prognostic value – at least in those patients who were not surgically treated according to the local algorithm. Associations between fracture characteristics and LOS are somewhat in between. LOS is influenced by the observed course of the patient as well as expectations about the future course and is still a clinical decision. In addition, the LOS distribution may be biased towards shorter durations, since a relevant proportion of patients was discharged early to a geriatric rehabilitation facility. However, it is unlikely that this biases the association with fracture characteristics.

We have defined individual fracture characteristics of pelvic ring fractures in elderly patients after low-energy trauma that support clinical decision making and promote patient-centred treatment algorithms. This includes an appreciation of the fracture morphology, but also patients’ baseline performance, risk-factors and outcome-expectations in form of a shared decision-making. Furthermore, we found that these fracture characteristics are also related to outcome. We acknowledge the limitations of our study. First, it is a retrospective analysis. Since follow up information was limited to survival, reliable data could be obtained from official records for almost all patients. Nevertheless, using overall survival as a measure probably only allows a glimpse of the tip of the iceberg and hides the true triggers and points for possible intervention. Rigorous follow-up assessments in terms of mobility and quality of life might provide better insights into the association of fracture characteristics with patient-relevant outcomes and change our judgment about the relevance of the five characteristics considered in this paper or help with the identification of additional characteristics.

## Conclusions

The assessment of the extent of dorsal fractures, the extent of the ventral fractures, the presence of a horizontal sacral fracture, a dislocated ventral fracture or a comminuted ventral fracture as specific morphological fracture characteristics of pelvic ring fractures in the elderly revealed associations with the decision for surgery, conservative treatment or failure of conservative treatment and the need for secondary surgical fracture stabilisation. In addition, LOS and survival were related to some of the fracture characteristics analysed. These characteristics thus represent a simplified but highly informative approach for the evaluation of pelvic ring fractures in the elderly. This approach can support clinical decision making, promote patient-centred treatment algorithms and thus improve the outcome of individualized care.

## Supplementary Information

**Additional file 1: Supplement 1.** Association of fracture characteristic with decision for surgery. Results from univariate and multivariate analyses

**Additional file 2: Supplement 2.** Association of fracture characteristic with length of stay. Results from univariate and multivariate analyses

**Additional file 3: Supplement 3.** Association of fracture characteristic with overall survival. Results from univariate and multivariate analyses

## Data Availability

The data that support the findings of this study has been compiled from various data sources in a pseudonymised fashion by some of the authors. The dataset is currently further evaluated. The source data are available from the University Hospital Basel but restrictions apply to the availability of these data, which were used under license for the current study, and so are not publicly available. Data are however available from the authors upon reasonable request within the applicable data protection laws (chapter 4 and 7 Human Research Act dated 30th of September 2011 and last updated of 26th of May 2021 and article 6, Data Protection Act dated 19th of June 1992 and last updated 1st of March 2019) and with permission of the University Hospital Basel.
