# Supplementary material for: Focusing on individual morphological fracture characteristics of pelvic ring fractures in elderly patients can support clinical decision making
Source: BMC Geriatr. 2022 Jun 30;22:543. doi: 10.1186/s12877-022-03222-0 (PMC9245220; doi:10.1186/s12877-022-03222-0)
Supplement: Supplementary file 1 — Additional file 1: Supplement 1. Association of fracture characteristic with decision for surgery. Results from univariate and multivariate analyses [file 12877_2022_3222_MOESM1_ESM.docx]

Supplement 1. Association of fracture characteristic with decision for surgery. Results from univariate and multivariate analyses

|  | unadjusted | | | adjusted | | |
| --- | --- | --- | --- | --- | --- | --- |
|  | OR | 95%-CI | p-value | OR | 95%-CI | p-value |
| Decision for surgery | | | | | | |
| extent of dorsal fractures | 7.7 | [4.8,12.4] | **< .001** | 7.0 | [3.8,13.0] | **< .001** |
| extent of ventral fractures | 0.9 | [0.5,1.5] | 0.601 | 1.0 | [0.6,1.6] | 0.905 |
| horizontal sacral fracture | 4.7 | [2.8,7.7] | **< .001** | 1.2 | [0.6,2.4] | 0.671 |
| dislocated ventral fracture | 2.5 | [1.5,4.0] | **< .001** | 2.4 | [1.3,4.2] | **0.004** |
| comminuted ventral fracture | 1.6 | [0.9,2.8] | 0.124 | 1.2 | [0.6,2.4] | 0.575 |
| Decision for early surgery | | | | | | |
| extent of dorsal fractures | 9.7 | [5.2,18.1] | **< .001** | 8.8 | [4.0,19.1] | **< .001** |
| extent of ventral fractures | 0.9 | [0.5,1.8] | 0.814 | 1.3 | [0.7,2.3] | 0.427 |
| horizontal sacral fracture | 5.5 | [3.0,10.0] | **< .001** | 1.3 | [0.6,3.0] | 0.487 |
| dislocated ventral fracture | 1.9 | [1.1,3.5] | **0.030** | 1.6 | [0.8,3.3] | 0.189 |
| comminuted ventral fracture | 1.0 | [0.4,2.2] | 0.985 | 0.8 | [0.3,1.9] | 0.579 |
| Rehospitalization for surgery | | | | | | |
| extent of dorsal fractures | 4.9 | [2.6,9.1] | **< .001** | 4.2 | [1.9,9.4] | **< .001** |
| extent of ventral fractures | 0.8 | [0.4,1.8] | 0.585 | 0.7 | [0.3,1.5] | 0.320 |
| horizontal sacral fracture | 3.2 | [1.5,6.6] | **0.002** | 0.9 | [0.3,2.5] | 0.851 |
| dislocated ventral fracture | 3.0 | [1.5,5.9] | **0.001** | 2.7 | [1.2,5.8] | **0.015** |
| comminuted ventral fracture | 2.3 | [1.1,4.9] | **0.027** | 1.7 | [0.7,4.0] | 0.227 |
