# Supplementary material for: Focusing on individual morphological fracture characteristics of pelvic ring fractures in elderly patients can support clinical decision making
Source: BMC Geriatr. 2022 Jun 30;22:543. doi: 10.1186/s12877-022-03222-0 (PMC9245220; doi:10.1186/s12877-022-03222-0)
Supplement: Supplementary file 2 — Additional file 2: Supplement 2. Association of fracture characteristic with length of stay. Results from univariate and multivariate analyses [file 12877_2022_3222_MOESM2_ESM.docx]

Supplement 2. Association of fracture characteristic with length of stay. Results from univariate and multivariate analyses

|  | unadjusted | | | adjusted | | |
| --- | --- | --- | --- | --- | --- | --- |
|  | OR | 95%-CI | p-value | OR | 95%-CI | p-value |
| Overall length of stay | | | | | | |
| extent of dorsal fractures | 2.2 | [1.7,2.8] | **< .001** | 1.8 | [1.4,2.5] | **< .001** |
| extent of ventral fractures | 1.2 | [0.9,1.7] | 0.268 | 1.2 | [0.8,1.7] | 0.298 |
| horizontal sacral fracture | 2.4 | [1.6,3.6] | **< .001** | 1.4 | [0.9,2.2] | 0.158 |
| dislocated ventral fracture | 2.0 | [1.4,2.8] | **< .001** | 1.7 | [1.2,2.4] | **0.003** |
| comminuted ventral fracture | 1.3 | [0.9,2.0] | 0.190 | 1.0 | [0.6,1.5] | 0.940 |
| Length of 1st stay in conservative | | | | | | |
| extent of dorsal fractures | 1.2 | [0.9,1.6] | 0.123 | 1.2 | [0.9,1.7] | 0.195 |
| extent of ventral fractures | 1.6 | [1.1,2.3] | **0.021** | 1.5 | [1.0,2.2] | 0.059 |
| horizontal sacral fracture | 1.1 | [0.7,1.7] | 0.651 | 0.9 | [0.5,1.5] | 0.660 |
| dislocated ventral fracture | 1.5 | [1.0,2.1] | **0.034** | 1.3 | [0.9,1.9] | 0.159 |
| comminuted ventral fracture | 1.2 | [0.8,1.8] | 0.403 | 1.0 | [0.6,1.6] | 0.969 |
