# Supplementary material for: Focusing on individual morphological fracture characteristics of pelvic ring fractures in elderly patients can support clinical decision making
Source: BMC Geriatr. 2022 Jun 30;22:543. doi: 10.1186/s12877-022-03222-0 (PMC9245220; doi:10.1186/s12877-022-03222-0)
Supplement: Supplementary file 3 — Additional file 3: Supplement 3. Association of fracture characteristic with overall survival. Results from univariate and multivariate analyses [file 12877_2022_3222_MOESM3_ESM.docx]

Supplement 3. Association of fracture characteristic with overall survival. Results from univariate and multivariate analyses

|  | unadjusted | | | adjusted | | |
| --- | --- | --- | --- | --- | --- | --- |
|  | HR | 95%-CI | p-value | HR | 95%-CI | p-value |
| overall survival | | | | | | |
| extent of dorsal fracture | 1.1 | [0.9,1.4] | 0.351 | 0.9 | [0.7,1.2] | 0.569 |
| extent of ventral fracture | 0.9 | [0.7,1.3] | 0.622 | 1.1 | [0.8,1.5] | 0.727 |
| horizontal sacral fracture | 1.5 | [1.1,2.2] | **0.014** | 1.5 | [1.0,2.2] | **0.048** |
| comminuted ventral fracture | 1.6 | [1.1,2.3] | **0.013** | 1.7 | [1.1,2.5] | **0.008** |
| dislocated ventral fracture | 1.3 | [1.0,1.7] | 0.102 | 1.2 | [0.8,1.6] | 0.361 |
